# Study on the Temperature and Load Dependence of Rutting Resistance for Large Stone Asphalt Mixture LSAM-50

**DOI:** 10.3390/ma19132731

**Published:** 2026-06-25

**Authors:** Ming Yang, Hong Li, Junhao Li, Chao Li, Yue Wang, Yingjun Jiang, Xiaolong Guo

**Affiliations:** 1 Henan Provincial Transportation Construction Technology Center, Zhengzhou 450018, China; mingy0401@163.com; 2 School of Highway, Chang’an University, Xi’an 710064, China; 2024221252@chd.edu.cn (J.L.); chaoli2026@163.com (C.L.); 2023021084@chd.edu.cn (Y.W.); jyj@chd.edu.cn (Y.J.); 3 Henan Yuxi Expressway Co., Ltd., Zhengzhou 450046, China; m19937528085@163.com

**Keywords:** large stone asphalt mixture, rutting resistance, temperature and load dependency, permanent deformation

## Abstract

To investigate the rutting resistance of Large Stone Asphalt Mixture (nominal maximum aggregate size of 53 mm, abbreviated as LSAM-50), this study evaluated the effects of temperature, load, and their interaction on the rutting performance of LSAM-50 through large-thickness rutting tests. It analyzed the characteristics of rutting deformation under varying thermal and loading conditions, established a permanent deformation-temperature-load dependency model, and explored the correlations between permanent deformation and high-temperature evaluation indicators. The findings indicate that the temperature-load interaction fundamentally alters the load-transfer mechanism between the viscoelastic matrix and coarse aggregates within LSAM-50, thereby activating the interlocking effect of its thick structural skeleton. The dynamic stability undergoes a pronounced reduction as temperature or load increases, peaking at a degradation rate of 40–57% within the 40–50 °C interval. Furthermore, the rutting deformation of the LSAM-50 mixture demonstrates significant temperature and load dependency; as the number of loading cycles increases, the deformation exhibits an initial rapid escalation before reaching a plateau. During temperature elevation and load escalation, the rutting deformation increases in a step-wise manner. Notably, the preliminary application of low temperatures and light loads imparts a substantial “training” effect on the material’s rutting resistance. Once the mixture is wheel-tracked to densification under high temperatures or heavy loads, negligible new deformation is generated during the subsequent cooling or unloading phases. Specifically, upon the initial unloading from 1.1 MPa to 0.9 MPa, the incremental deformation is merely 0.04 mm; upon further unloading to 0.7 MPa, the additional deformation approaches 0 mm. The established permanent deformation-temperature-load dependency model for LSAM-50 yields a high predictive correlation of 96%. Moreover, the permanent deformation exhibits robust linear relationships with 1-h rutting depth (*R*^2^ = 0.95), compressive strength (*R*^2^ = 0.91), and shear strength (*R*^2^ = 0.97). These indicators can thus facilitate the rapid and precise estimation of permanent pavement deformation.

## 1. Introduction

Flexible base courses exhibit distinct advantages in optimizing pavement structural stress distribution and extending service life, owing to their more moderate stress response under low-temperature conditions, superior interlayer bonding, more coordinated modulus transition across structural layers, and effective mitigation of reflection cracking [1,2]. However, in practical engineering applications, typical flexible base materials (e.g., ATB-30) remain susceptible to rutting accumulation and subsequent structural damage under the long-term coupling effects of high-temperature environments and heavy traffic loads. Furthermore, their material and construction costs are relatively high [3,4]. To address these deficiencies, researchers have conducted extensive investigations into binder performance enhancement and mixture structural optimization, such as employing polymer modification technologies to improve the high-temperature deformation resistance of asphalt, or adjusting gradations to enhance skeleton structural stability [5,6]. In recent years, Large Stone Asphalt Mixture with a nominal maximum aggregate size of 53 mm (LSAM-50) has emerged as a significant research focal point. Relying on a load-bearing structure dominated by coarse aggregates, this material not only possesses higher overall strength but also significantly reduces the required asphalt content due to the decreased specific surface area of the mineral aggregates, thereby ensuring economic feasibility while enhancing high-temperature stability. Moreover, it facilitates single-layer thick-lift paving, further improving construction efficiency and structural integrity [7,8,9]. Compared with traditional ATB materials, LSAM-50 demonstrates a superior balance between mechanical performance and cost control, offering substantial value for engineering promotion and application.

To simulate the effects of traffic loading on asphalt pavements, scholars have developed both mechanistic and empirical testing methods. Mechanistic testing methods acquire stress-strain relationships to evaluate the permanent deformation performance of mixtures, whereas empirical testing methods simulate wheel loading to test mixture deformation, directly reflecting rutting resistance. Although numerous high-temperature performance testing methods for asphalt mixtures, such as creep tests [10,11,12], shear tests [13], and simple performance tests [14], have been developed and applied, the wheel tracking test remains the most widely utilized method. The wheel tracking test has been proven to effectively simulate the repetitive action of loads on pavements, and can model the impacts of temperature, load, asphalt layer thickness, compaction method, and moisture on pavement rutting deformation [15,16]. However, the wheel tracking test cannot yet obtain the mechanical parameters of asphalt mixtures, and its results are rarely employed in theoretical calculations [17]. Based on measured data, Monismith utilized statistical analysis to derive a rutting prediction equation with temperature, stress, loading time, and mixture volumetric parameters as variables [18]. Wijevatne conducted triaxial tests on asphalt mixture specimens; the results indicated that specimen deformation and loading exhibited a strong linear relationship on a log-log plot, based on which an empirical formula between permanent deformation, material parameters, and the number of loading cycles was established [19]. Through laboratory APA tests, the Shami model proposed a rutting deformation prediction equation using temperature and the number of axle load applications as independent variables [20]. Sides constructed a constitutive model comprehensively considering the visco-elasto-plasticity of asphalt materials; utilizing the finite element method, they discovered that when the temperature exceeds 25 °C, rutting deformation is primarily composed of plastic and visco-plastic strains [21]. Based on Perzyna’s viscoplastic theory combined with the Drucker-Prager yield failure criterion, Park established a visco-elasto-plastic model for asphalt mixtures under one-dimensional conditions [22]. Whiffin introduced the TRRL model developed by the UK Transport and Road Research Laboratory (TRRL), which accounts for the number of axle load applications and the deviatoric stress of the asphalt mixture to calculate the permanent deformation of pavements [23]. Based on measured rutting data from 61 test tracks, Ali established a Mechanistic-Empirical (M-E) rutting prediction model using the vertical strain of each structural layer of the pavement as the control indicator [24]. Utilizing pavement material parameters and rutting test data obtained from WesTrack test sections, Hand pioneered the consideration of material shear stress within rutting prediction models [25]. Javilla conducted rutting tests on AC-20 limestone and AC-13 basalt, discovering that the rutting deformation generated after 1000 loading cycles exhibited an exponential functional relationship with long-term rutting development [26]. Qadir demonstrated, based on a developed linear regression model, that the rutting behavior of asphalt mixtures could be effectively depicted through a linear model of pavement temperature and polymer type [27]. Currently, there is a limited amount of research related to the rutting resistance of large stone asphalt mixtures, involving testing methods such as high-temperature creep tests, wheel tracking tests, and uniaxial penetration tests. Hugo [28], Fernando [29], and others employed high-temperature creep tests to evaluate the rutting resistance of large stone asphalt mixtures. Researchers such as Mohammad [30], Horak [31], Coree [32], Park [33], and Mascarenhas [34] utilized laboratory wheel tracking tests to evaluate the rutting resistance of large stone asphalt mixtures. The nominal maximum aggregate sizes included 26.5 mm, 31.5 mm, and 37.5 mm, with wheel tracking specimen thicknesses of 8 cm, 10 cm, and 15 cm. Their research indicated that large stone mixtures possess superior rutting resistance compared to traditional asphalt mixtures. Furthermore, increasing the thickness of the asphalt mixture wheel tracking specimens is more advantageous regarding the coefficient of variation of the test results, offering enhanced repeatability.

The aforementioned research outcomes provide valuable references for evaluating the rutting resistance of LSAM-50. However, existing studies lack a comprehensive analysis of the temperature and load dependencies concerning the rutting resistance of the LSAM-50 asphalt mixture. Furthermore, there is a scarcity of research exploring the impacts of complex temperature variation paths and loading histories on its structural stability. In light of these research gaps, this study quantifies the coupling effects of temperature and load on the deformation resistance of LSAM-50 via full-factor, large-thickness wheel tracking tests. It delves into the developmental and evolutionary characteristics of rutting under varying temperature paths and staged loading histories. Subsequently, grounded in macroscopic experimental phenomena and mechanical boundary conditions, a high-precision permanent deformation-temperature-load dependency prediction model for LSAM-50 is established. The findings of this research will furnish robust theoretical support for the scientific design, construction application, and long-term performance evaluation of LSAM-50 thick-lift flexible base courses.

## 2. Materials and Methods

### 2.1. Materials

(1) Base asphalt

The asphalt binder utilized in this study was Zhenhai 70# heavy-traffic paving petroleum asphalt. The technical properties of the asphalt were evaluated in accordance with the Standard Test Methods of Bitumen and Bituminous Mixtures for Highway Engineering (JTG 3410—2025) [35], and the specific technical indicators are detailed in Table 1.

(2) Aggregates

The aggregates utilized in this study comprised crushed limestone and manufactured sand sourced from Baoji, Shaanxi Province. The technical properties of these aggregates are detailed in Table 2.

(3) Mineral Filler

The mineral filler utilized in this research was limestone filler sourced from Xianyang, Shaanxi Province. Its apparent specific gravity and moisture content were 2.713 and 0.4%, respectively.

(4) Mineral Aggregate Gradation

**Figure 1:**
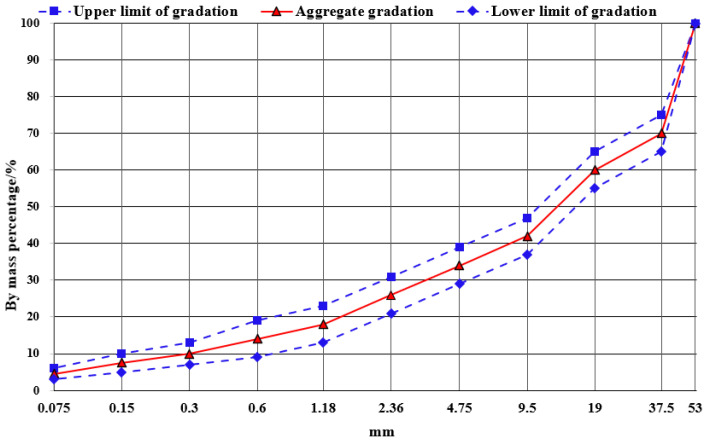
Aggregate gradation curve.

The experimental aggregate gradation is presented in Table 3, with its corresponding gradation curve plotted in Figure 1.

### 2.2. Methods

(1) Large-Thickness Rutting Test

**Figure 2:**
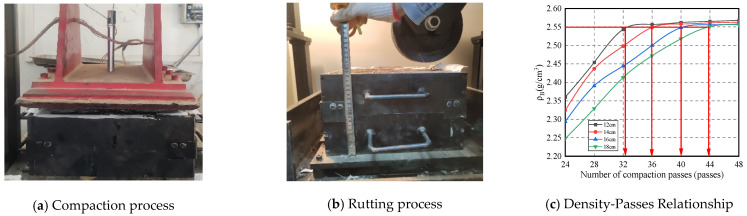
Large-thickness rutting test.

The large-thickness wheel tracking specimens of the LSAM-50 asphalt mixture were fabricated utilizing a specialized roller compactor (TDCX-2, Cangzhou Hengsheng Weiye Highway Instrument Co., Ltd., Cangzhou, China) designed for thick-lift specimens. The dimensions of the specimens were 300 mm (length) × 300 mm (width) × 160 mm (height). The influence of the number of compaction passes on the bulk density (ρ_B_) of wheel tracking slabs across various thicknesses is detailed in Table 4 and illustrated in Figure 2. The optimal number of compaction passes was determined to be 40, which enables the mixture to achieve a densification level comparable to that of in-situ field compaction. All wheel tracking tests were conducted employing a large-thickness wheel tracking testing apparatus. To ensure a uniform internal temperature distribution within the specimens, distinct thermal conditioning durations were established based on the target testing temperatures: 2.5 h at 20 °C, 3 h at 30 °C, 4 h at 40 °C, 6 h at 50 °C, and 7 h at 60 °C. Under each experimental condition, three replicate specimens were prepared and tested, with the final reported results representing the average of the three trials. The experimental procedure for the large-thickness wheel tracking test is depicted in Figure 2.

(2) Constant Temperature and Constant Load Test

To analyze the impacts of temperature and load on the rutting resistance of the LSAM-50 asphalt mixture and to establish a permanent deformation-temperature-load dependency model, wheel tracking tests were conducted under constant temperature and loading conditions. To comprehensively encompass the actual in-service temperature range of LSAM-50 within pavement base courses and to align with current testing standards, five testing temperature levels were established: 20 °C, 30 °C, 40 °C, 50 °C, and 60 °C. Concurrently, to simulate diverse traffic loading conditions ranging from light traffic to extreme overloading, four load levels were selected: 0.5 MPa, 0.7 MPa, 0.9 MPa, and 1.1 MPa, thereby constituting a full-factorial experimental design. In accordance with the Standard Test Methods of Bitumen and Bituminous Mixtures for Highway Engineering (JTG 3410—2025), the loading rate for the wheel tracking test was set at 42 ± 0.5 passes/min. Considering the prolonged densification process of the LSAM-50 asphalt mixture attributable to its large aggregate skeleton size, each test group was subjected to continuous loading for 7650 passes (equivalent to a duration of 3 h), with the rutting depth recorded in real time. For every temperature-load scenario within the full-factorial matrix, three replicate tests were performed, and the final results represent the average of these three trials.

(3) Varying Temperature Path Test

In actual in-service environments, the cumulative deformation response of pavements is highly dependent on their experienced temperature and loading histories. To investigate the impacts of temperature variation paths on the rutting resistance of the LSAM-50 asphalt mixture, tests were conducted employing two distinct thermal variation modes: stepwise temperature elevation and stepwise temperature reduction. Five temperature levels were established: 20 °C, 30 °C, 40 °C, 50 °C, and 60 °C. Under the stepwise temperature elevation mode, specimens were initially thermally conditioned at the lowest temperature and subjected to 7650 loading passes. Upon pausing the test, the environmental temperature was elevated to the subsequent level; the specimens were then re-conditioned for 7 h prior to being subjected to another 7650 loading passes. This sequence was repeated iteratively until the loading across all five temperature levels was completed. Conversely, the stepwise temperature reduction mode commenced at the highest temperature and progressively decreased to the lowest temperature level following a corresponding procedure. For both the heating and cooling paths, three replicate tests were conducted, with the final reported results representing the average of the three trials.

(4) Varying Load Path Test

To investigate the impacts of loading history on the rutting resistance of the LSAM-50 asphalt mixture, tests were conducted employing two distinct varying-load modes: stepwise loading and stepwise unloading. Four load levels were established: 0.5 MPa, 0.7 MPa, 0.9 MPa, and 1.1 MPa. Under the stepwise loading mode, specimens were initially subjected to the lowest load for 7650 passes. Upon pausing the test, the applied load was elevated to the subsequent level, and the specimens were subjected to an additional 7650 loading passes. This sequence was repeated iteratively until the loading across all four levels was completed. Conversely, the stepwise unloading mode commenced at the highest load and progressively decreased to the lowest load level following a corresponding procedure. For each varying-load mode, three replicate tests were conducted, with the final reported results representing the average of the three trials.

## 3. Study on Factors Influencing the Rutting Resistance of LSAM-50 Asphalt Mixture

### 3.1. Factors Influencing the Dynamic Stability of LSAM-50 Asphalt Mixture

(1) Effect of Temperature

**Figure 3:**
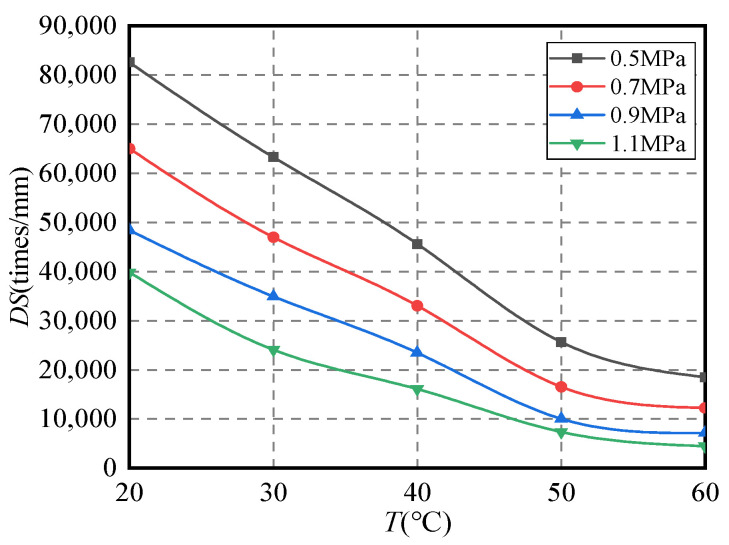
Relationship between dynamic stability and temperature.

**Figure 4:**
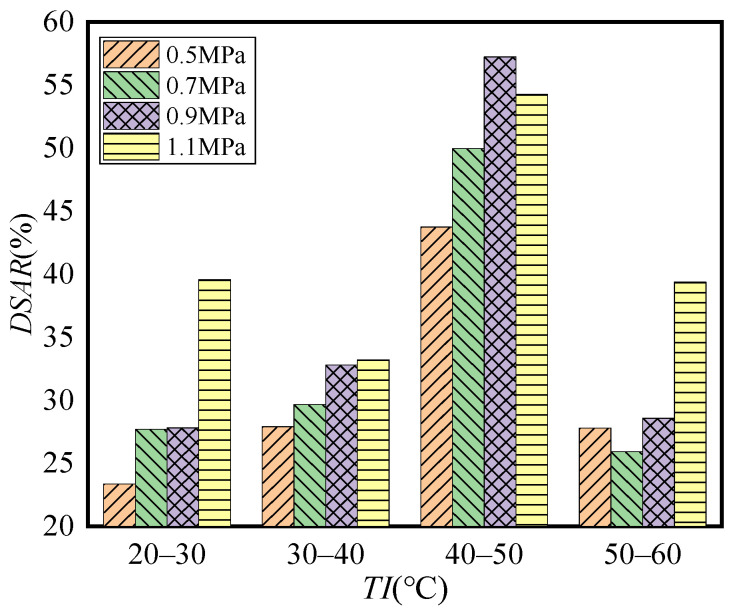
Dynamic stability attenuation rate in different temperature intervals.

The variation in the dynamic stability of the LSAM-50 asphalt mixture with respect to temperature is illustrated in Figure 3, while the dynamic stability attenuation rate across different temperature intervals is presented in Figure 4. In these figures, T denotes temperature, DS represents dynamic stability, DSAR indicates the dynamic stability attenuation rate, and TI stands for the temperature interval. Dynamic Stability (DS) is defined as the number of wheel tracking passes required to generate 1 mm of deformation in the asphalt mixture under high-temperature conditions, serving as a metric to evaluate the material’s resistance to permanent deformation. The Temperature Interval (TI) is defined as the range between two adjacent testing temperature points. Furthermore, the Dynamic Stability Attenuation Rate (DSAR) is utilized to quantify the degree of stability loss exhibited by the mixture within a specific temperature interval.

As illustrated in Figure 3, the dynamic stability of the LSAM-50 asphalt mixture across all load levels exhibits a pronounced declining trend with increasing temperature. This observation indicates that the high-temperature rutting response of the material is highly temperature-sensitive; essentially, elevated temperatures consistently result in a diminished capacity of the mixture to resist permanent deformation.

As shown in Figure 4, the dynamic stability attenuation rate (DSAR) of the LSAM-50 asphalt mixture across different load levels follows a consistent trend, increasing initially and subsequently decreasing as temperature rises. Specifically, the DSAR values within the intervals of 20–30 °C, 30–40 °C, 40–50 °C, and 50–60 °C are 23–40%, 28–33%, 40–57%, and 26–39%, respectively. The DSAR reaches its peak within the 40–50 °C interval, which corresponds to the proximity of the asphalt softening point. This peak can be attributed to the phase transition occurring within the asphalt mastic from a high-elastic state to a viscous-flow state as the temperature approaches the softening point. During this process, the expansion of the free volume of molecular chains leads to a sharp decline in the cohesive strength of the mastic. At this stage, the asphalt mastic not only functions as a binder but also induces a physical lubrication effect due to the reduction in viscosity, which diminishes the frictional resistance between coarse aggregate skeletons. Macroscopically, this manifests as a weakened resistance to shear deformation. These observations suggest that the properties of the LSAM-50 asphalt mixture undergo significant alterations near the asphalt softening point. Furthermore, within the same temperature interval, the DSAR increases progressively with the escalation of the applied load.

(2) Effect of Load

**Figure 5:**
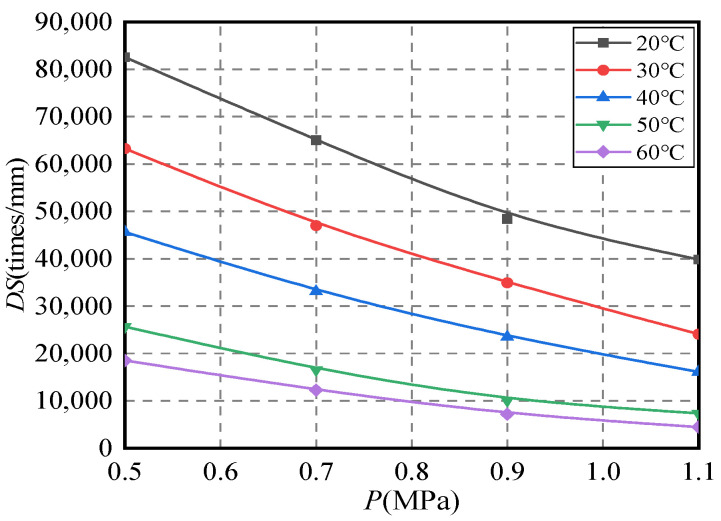
Relationship between dynamic stability and load.

**Figure 6:**
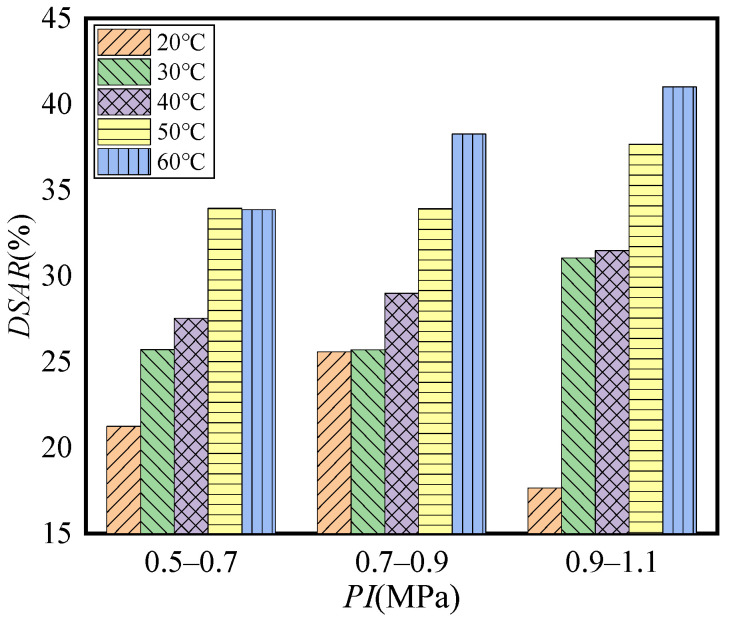
Dynamic stability attenuation rate in different load intervals.

The variation law of the dynamic stability of the LSAM-50 with load is shown in Figure 5, and the dynamic stability attenuation rates within different load ranges are shown in Figure 6. In the figures, *P* denotes the load, and *PI* denotes the load interval.

From Figure 5, it can be observed that as the load increases, the dynamic stability of the LSAM-50 asphalt mixture under various temperatures decreases significantly. The increase in wheel load leads to a further increase in the deformation of the mixture.

From Figure 6, it can be seen that: when the temperature is higher than 20 °C, the dynamic stability attenuation rate generally displays a monotonically increasing trend with the increase in load; in the intervals of 0.5–0.7 MPa, 0.7–0.9 MPa, and 0.9–1.1 MPa, the rates are 21–34%, 25–38%, and 17–41%, respectively. When the temperature is 20 °C, the dynamic stability attenuation rate exhibits an upward convex parabolic trend with increasing load, reaching its peak in the 0.7–0.9 MPa interval. Within each load interval, the dynamic stability attenuation rate displays a monotonically increasing trend with rising temperature, indicating that high temperatures further amplify the destructive effect of loads on asphalt pavements. That is, high temperatures and heavy loads have a coupled effect on pavement deformation.

(3) Coupled Effect of Temperature and Load

To quantitatively evaluate the coupling effects of temperature and load on the rutting resistance of the LSAM-50 asphalt mixture, a two-way analysis of variance (ANOVA) was performed to conduct statistical testing on the dynamic stability (DS) data. In this analytical model, “Temperature” and “Load” were designated as two independent factors, comprising five and four levels, respectively. Each full-factorial combination of temperature and load included three replicate tests, resulting in a total sample size of *n* = 60.

Within this statistical framework, the physical quantities and statistical indicators are defined as follows: the Sum of Squares (SS) represents the total variation in the data attributable to the corresponding factors or random errors; the Degrees of Freedom (df) denotes the number of independent pieces of information used to calculate each statistic; the Mean Square (MS) is derived by dividing the SS by its corresponding df, representing the variance per degree of freedom; and the F-value constitutes the test statistic, calculated as the ratio of the MS of each factor to the error MS. In this study, F*_T_*, F*_P_*, and F*_I_* represent the calculated statistics for temperature, load, and their interaction, respectively. The results of the two-way ANOVA are detailed in Table 5.

Setting the significance level at a = 5%, F_0.05_(4, 40) = 2.61, F_0.05_(3, 40) = 2.84, F_0.05_(12, 40) = 2.00, comparisons reveal F*_T_* > 2.61, F*_P_* > 2.84, and F*_I_* > 2.00. According to the results of the two-way ANOVA, both temperature and load have significant effects on the dynamic stability of the LSAM-50 asphalt mixture; concurrently, there is a significant interactive effect between the two on dynamic stability.

### 3.2. Effect of Temperature and Load on Rutting Resistance of LSAM-50 Asphalt Mixture

The relationship between the rutting deformation of the LSAM-50 asphalt mixture and the number of load applications is shown in Figure 6. In the figure, *RD* denotes the amount of rutting deformation, and *N* denotes the number of load applications.

**Figure 7:**
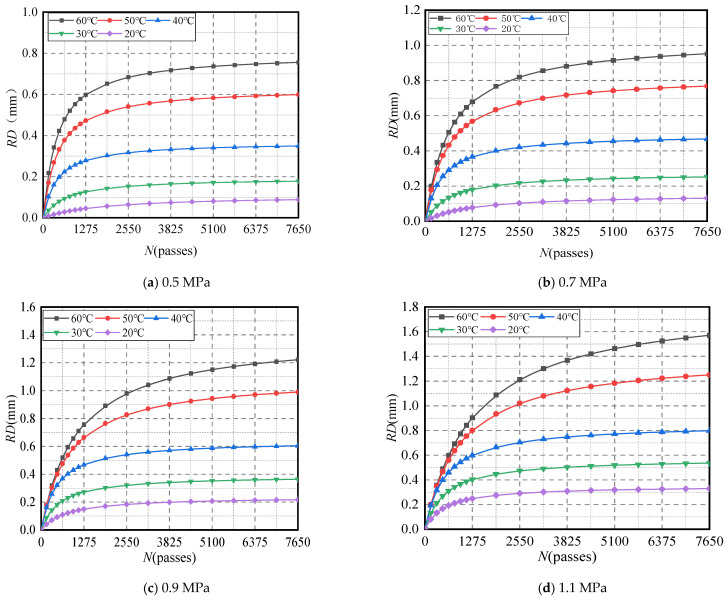
Relationship between rutting deformation of LSAM-50 asphalt mixture and the number of load applications.

From Figure 7, it can be concluded that:

① As the number of load applications increases, the rutting development curves of the LSAM-50 asphalt mixture at different temperatures under each load are highly similar. In the initial stage of loading, the air void content of the mixture is relatively large. Under external forces, the coarse aggregates gradually densify to form a skeleton structure, and the asphalt mastic and fine aggregates are rapidly squeezed into the gaps between the mineral aggregates, leading to a sharp increase in the rutting depth of the LSAM-50 asphalt mixture. As the number of load applications continues to increase, the air void content of the mixture gradually decreases, and the specimen becomes further densified after compaction. The high-temperature deformation resistance of the LSAM-50 asphalt mixture improves somewhat, and the rutting depth undergoes an approximately linear change. When the voids within the skeleton are filled to saturation, the rate of increase in rutting depth diminishes and tends to stabilize. This indicates that the rutting deformation of the LSAM-50 asphalt mixture approaches a limit value *RD*_∞_, as the number of load applications increases.

② As the temperature rises, the rutting depth of the LSAM-50 asphalt mixture increases significantly; this is because the temperature rise causes the modulus of the LSAM-50 asphalt mixture to continuously decrease, and the deformation response generated under the same load further increases. Considering that when the temperature is low and traffic loads are sparsely distributed, the asphalt mixture is in an elastic state, the generated rutting amount is negligible compared to the rutting during high-temperature, heavy-load periods.

③ In actual pavements, the temperature below which the rutting deformation contributes less than 10% to the total rutting is defined as the critical temperature. Based on this, taking the rutting amount generated by the specimen tracking for 3 h under standard conditions of 60 °C and 0.7 MPa as the baseline, when the rutting generated by the LSAM-50 asphalt mixture tracking for 3 h at a certain temperature is less than 10% of the baseline value, this temperature is considered the rutting critical temperature under that specific load level. According to Figure 6, the rutting critical temperatures corresponding to 0.5 MPa, 0.7 MPa, 0.9 MPa, and 1.1 MPa are 20 °C, 17 °C, 10 °C, and 7 °C, respectively. As the load increases, the critical temperature progressively decreases.

### 3.3. Effect of Varying Temperature Paths on Rutting Resistance of LSAM-50 Asphalt Mixture

(1) Effect of the Heating Process

**Figure 8:**
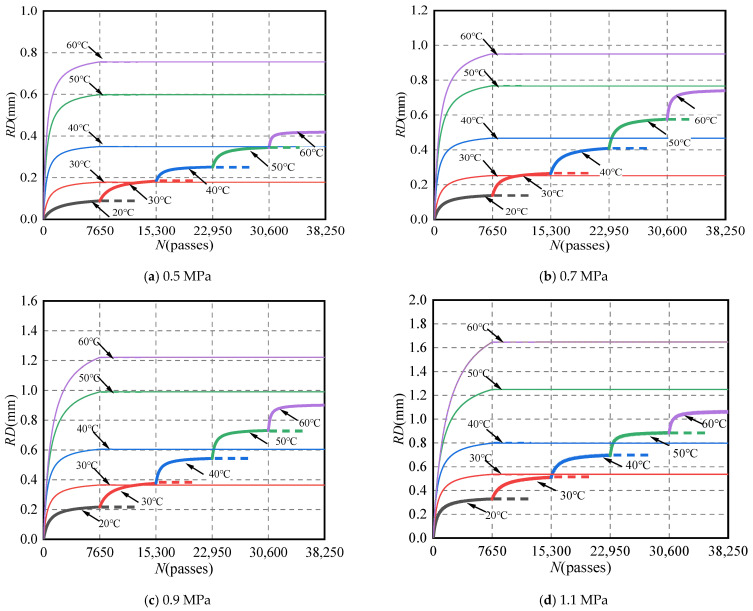
Rutting deformation of LSAM-50 asphalt mixture under heating and constant temperature modes.

The rutting deformation behavior of the LSAM-50 asphalt mixture during step-up heating and constant temperature processes is depicted in Figure 8.

From Figure 8, it can be seen that after heating, the deformation curve of the LSAM-50 asphalt mixture remains consistent with that before heating, both consisting of a densification deformation stage and a deformation stabilization stage. In the initial loading phase, the rutting depth grows rapidly; after a certain number of load applications, the degree of compaction gradually increases, and the deformation growth rate gradually slows down and approaches zero in the later stages. Furthermore, as the temperature increases, the number of load applications required to reach the deformation stabilization stage gradually decreases. Additionally, during the step-up heating process, the rutting deformation of the LSAM-50 asphalt mixture is consistently less than the deformation of the mixture when reaching this temperature during a constant temperature process.

**Figure 9:**
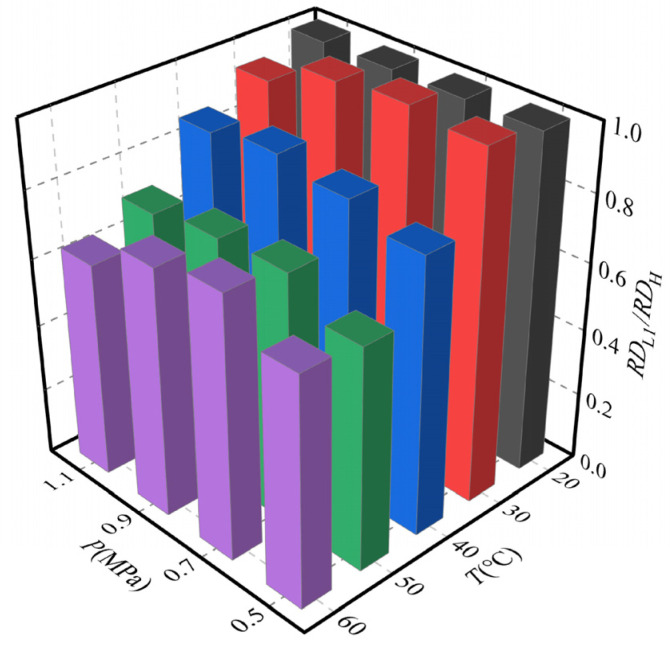
Relationship between *RD*_L1_/*RD*_HW_ of LSAM-50 asphalt mixture and temperature/load.

Table 6 details the incremental permanent deformation (*RD_S_*), the cumulative permanent deformation from preceding stages (*RD_L1_*), and the permanent deformation under constant temperature (*RD_HW_*) for the LSAM-50 asphalt mixture during the temperature elevation process. Accordingly, the ratio *RD_L1_/RD_HW_* is plotted in Figure 9. Within this context, *RD_S_* denotes the incremental permanent deformation generated within a specific temperature level under the varying temperature path; *RD_L1_* signifies the cumulative permanent deformation accumulated from the prior temperature stages up to the current level; and *RD_HW_* represents the permanent deformation of the mixture under standard testing conditions (i.e., at a single constant temperature).

From Figure 9, it can be seen that:

① The permanent deformation of the mixture under each constant temperature condition is consistently higher than the deformation accumulated when reaching the corresponding temperature under the temperature elevation path. This observation indicates that the permanent deformation of the LSAM-50 asphalt mixture exhibits a significant “training effect” during the temperature elevation process. In contrast to the work hardening induced by internal dislocations in metallic materials, the “training effect” in asphalt mixtures primarily stems from their visco-elasto-plastic characteristics and multiphase composite structure. During the lower-temperature stages, the asphalt mastic maintains a higher stiffness, and the external load effectively facilitates the secondary compaction of the mixture. This process induces the microscopic rearrangement and rotation of the large crushed stone aggregates within LSAM-50, further compressing the air voids and forging a more robust aggregate interlocking skeleton network. This preliminary densification action elevates the macroscopic yield stress of the material and mitigates the initiation of internal microcracks. Consequently, when the temperature escalates to the subsequent level, the “trained” mixture already possesses a superior stress distribution state and an enhanced resistance to shear rheology. This results in a permanent deformation under equivalent high-temperature conditions that is substantially lower than that of a mixture which has not experienced the preceding low-temperature actions.

② During the step-up heating process, the ratio of cumulative heating deformation to constant temperature deformation gradually decreases with increasing temperature and tends to stabilize. At 60 °C, the ratio between the two is approximately 70%.

(2) Effect of the Cooling Process

**Figure 10:**
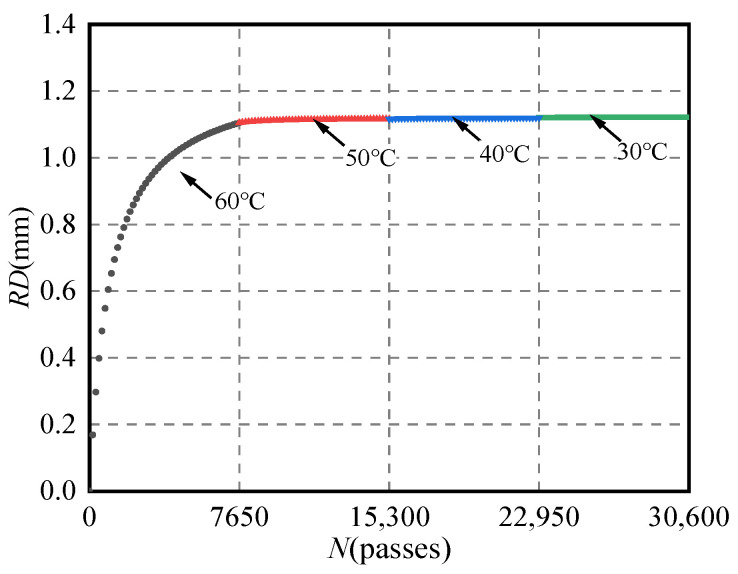
Rutting deformation of LSAM-50 asphalt mixture under cooling mode.

The rutting deformation of the LSAM-50 asphalt mixture during the cooling process is shown in Figure 10, with a load of 0.7 MPa.

From Figure 9, it can be seen that: during the step-down cooling process, the LSAM-50 asphalt mixture in a low-temperature state generates almost no deformation under loading. This is because the mixture has already reached a dense state under high temperature and load applications; moreover, as the temperature decreases, the modulus of the LSAM-50 asphalt mixture rises significantly, augmenting its deformation resistance. Therefore, the deformation during the cooling phase depends primarily on the rutting deformation of the mixture at the initial temperature.

### 3.4. Effect of Loading History on Rutting Resistance of LSAM-50 Asphalt Mixture

(1) Effect of the Loading Process

**Figure 11:**
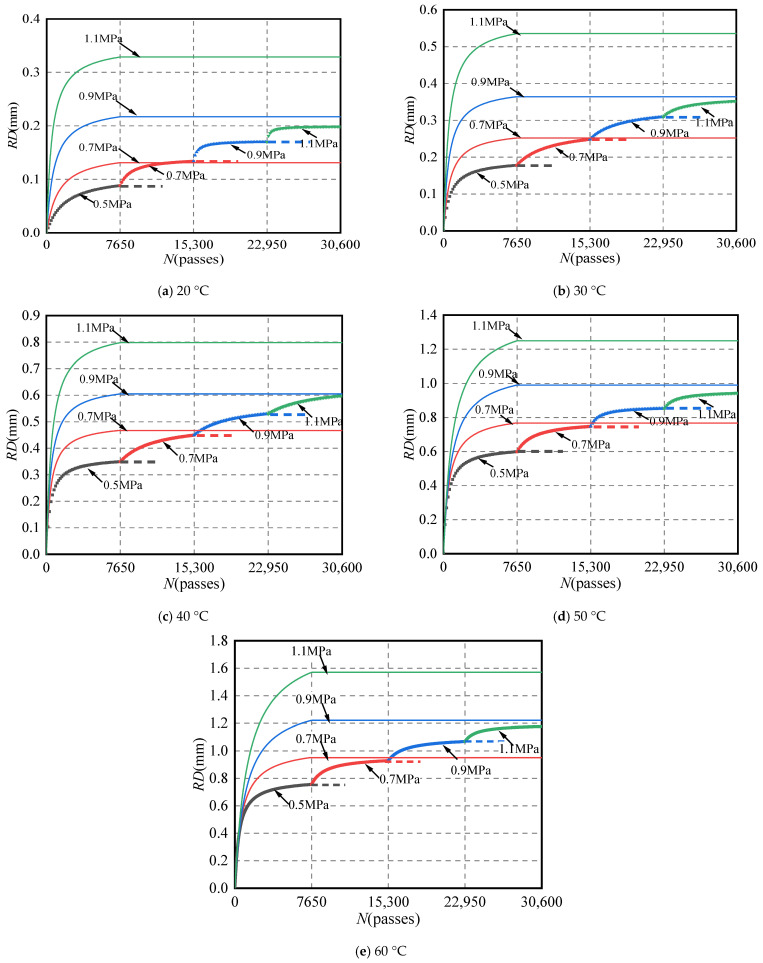
Rutting deformation of LSAM-50 asphalt mixture under loading and constant load modes.

The rutting deformation of the LSAM-50 asphalt mixture during the step-up loading and constant load processes is shown in Figure 11.

From Figure 11, it can be seen that:

① Similar to the heating phase, the deformation curve of the LSAM-50 asphalt mixture after an increase in load also consists of a densification deformation stage and a deformation stabilization stage, with the rate of deformation growth gradually decelerating and approaching zero. This indicates that increasing the load and elevating the temperature possess a certain degree of equivalent effect. As the load increases, deformation will further increase provided the mixture modulus remains unchanged.

② During the step-up loading process, the rutting deformation of the LSAM-50 asphalt mixture is consistently less than the deformation of the mixture upon reaching this load during a constant load process.

**Figure 12:**
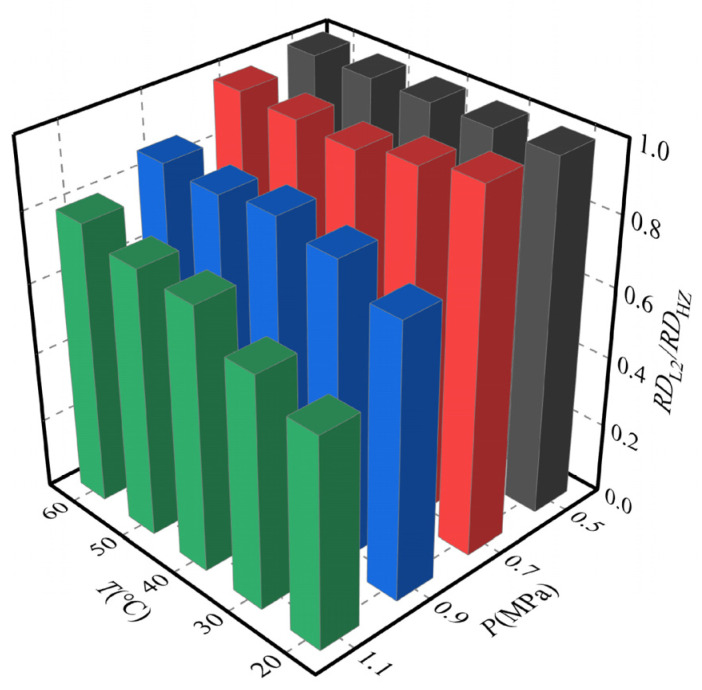
Relationship between *RD*_L2_/*RD*_HZ_ of LSAM-50 asphalt mixture and temperature/load.

Table 7 details the incremental permanent deformation (*RD_Z_*), the cumulative permanent deformation from preceding load stages (*RD_L2_*), and the permanent deformation under constant load (*RD_HZ_*) for the LSAM-50 asphalt mixture during the stepwise loading process. Accordingly, the ratio *RD_L2_/RD_HZ_* is plotted in Figure 12. Within this context, *RD_Z_* denotes the incremental permanent deformation generated within a specific load level under the varying-load path; *RD_L2_* signifies the cumulative permanent deformation accumulated from the prior load stages up to the current level; and *RD_HZ_* represents the permanent deformation of the mixture under standard testing conditions (i.e., at a single constant load).

From Figure 12, it can be seen that:

① The permanent deformation of the LSAM-50 asphalt mixture under each constant load application is higher than the permanent deformation of the mixture upon reaching this load under variable loading conditions. Consistent with the heating mode, a “training effect” also exists in the permanent deformation of the LSAM-50 asphalt mixture during the loading process. After experiencing lower loads, the wheel track zone of the LSAM-50 asphalt mixture undergoes a certain degree of deformation and becomes more compact. At this juncture, its mechanical properties have altered, and its ability to resist deformation has improved.

② The amount of deformation generated by each level of load during the loading process gradually decreases, indicating that the load history previously experienced by the asphalt mixture significantly impacts later rutting development. To a certain extent, it can mitigate the rutting deformation of the LSAM-50 asphalt mixture under subsequent loading.

③ During the step-up loading process, *RD*_L2_/*RD*_HZ_ gradually decreases as the load increases. The deformation generated when loading from 0.5 MPa to 0.7 MPa is relatively close to that under a constant load of 0.7 MPa. At 1.1 MPa, *RD*_L2_/*RD*_HZ_ is approximately 70%.

(2) Effect of the Unloading Process

**Figure 13:**
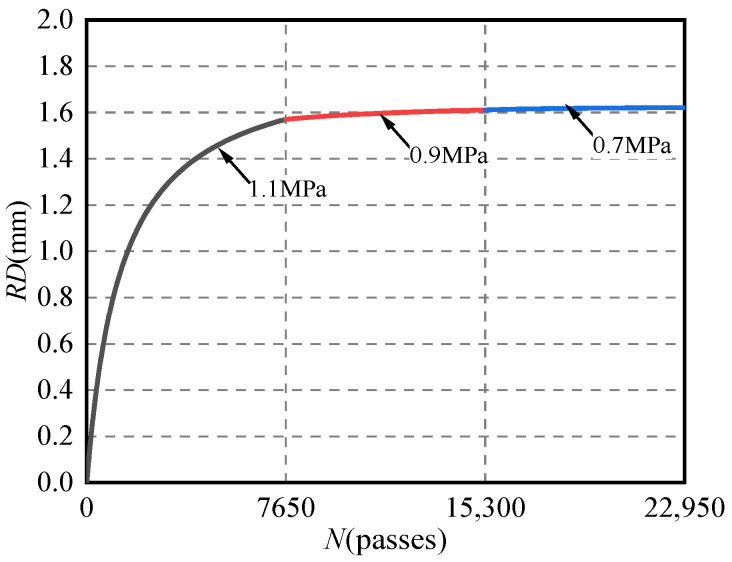
Rutting deformation of LSAM-50 asphalt mixture under unloading mode.

The rutting deformation of the LSAM-50 asphalt mixture during the unloading process is shown in Figure 13, at a temperature of 60 °C.

From Figure 13, it can be seen that: during the step-down unloading process, the LSAM-50 asphalt mixture under a light load state generates almost no deformation upon load application. This is because the mixture has already reached a dense state under heavy loading and load cycles, significantly improving its deformation resistance.

## 4. Study on Temperature and Load Dependence Model of Rutting Resistance for LSAM-50 Asphalt Mixture

### 4.1. Temperature and Load Dependency Model of Permanent Deformation

(1) Rutting Deformation Growth Equation

The permanent deformation of asphalt mixtures constitutes a quintessential process of visco-elastic and visco-plastic accumulation. For an ultra-large aggregate material with a coarse skeleton structure such as LSAM-50, its deformation under wheel loading primarily originates from the initial compaction of air voids and the subsequent restructuring of the aggregate skeleton. As the number of loading cycles increases, a stable and dense interlocking network gradually forms among the coarse aggregate particles. Corroborated by the macroscopic experimental results presented previously in Figure 7, it is clearly observable that following an initial phase of rapid deformation, the rutting development curves under all temperature and loading conditions decelerate significantly and gradually plateau. This phenomenon substantiates that the internal skeletal resistance to deformation has progressively attained a dynamic equilibrium with the external deviatoric stress. Consequently, unlike traditional empirical prediction models characterized by unbounded growth, the rutting development curve of the LSAM-50 asphalt mixture asymptotically approaches a limit value. This limit represents the ultimate permanent deformation, denoted as RD_∞_, under the specific temperature and loading conditions. Accordingly, the rutting deformation growth equation for the LSAM-50 asphalt mixture must satisfy the two boundary conditions stipulated in Equation (1):

(1) $$\begin{array}{l} N=0,\;RD(0)=RD_{0}=0\mathrm{mm}\\ N=\infty ,\;RD(\infty )=RD_{\infty } \end{array}$$

where *N* represents the number of load applications (times), *RD*(0) represents the initial rutting depth of the LSAM-50 asphalt mixture (mm), and *RD*(∞) represents the permanent deformation of the LSAM-50 asphalt mixture (mm).

Based on the above boundary conditions, the rutting deformation growth equation for the LSAM-50 asphalt mixture is established, as shown in Equation (2).

(2) $$RD(N)=\frac{RD_{\infty }\times N}{N+\xi }$$

where *ξ* represents the regression coefficient, which is related to the load.

Substituting the deformation amounts under different numbers of action cycles into Equation (2), regression analysis yields the rutting deformation growth equation for the LSAM-50 asphalt mixture. The relevant parameters are shown in Table 7.

From Table 8, it can be observed that the correlations between the fitted curves and the measured deformation curves are all above 0.9, indicating that this equation can accurately characterize the law of rutting development.

(2) Temperature and Load Dependency Model of Permanent Deformation

According to Table 8, the temperature and load dependency model of permanent deformation must satisfy the boundary conditions of Equation (3).

(3) $$\begin{array}{l} T<T_{0},\;RD_{\infty }\approx 0;\\ T_{1}<T_{2},\;0\leq RD_{T1}\leq RD_{T2} \end{array}$$

The rutting deformation of asphalt mixtures is directly governed by the viscous resistance of the internal binder, and the attenuation of this resistance with temperature typically follows the classic Arrhenius exponential law. For a coarse-skeleton mixture such as LSAM-50, when the ambient temperature is below a specific critical threshold (referred to as the critical rutting temperature, *T*_0_), the high-viscosity asphalt acts in synergy with the coarse aggregates to provide extremely high shear stiffness, resulting in negligible macroscopic permanent deformation. However, once the temperature exceeds *T*_0_, the binder softens abruptly, and the lubrication effect between skeleton particles is intensified, leading to an exponential, non-linear increase in permanent deformation as temperature rises. Based on these thermodynamic rheological mechanisms, and to satisfy the mathematical requirement that the theoretical deformation remains consistently positive as well as the boundary conditions stipulated in Equation (3), this study develops an exponential evolution model incorporating the critical temperature parameter, as formulated in Equation (4).

(4) $$RD_{\infty (T)}=A\times EXP(-\left|\frac{B}{T_{0}-\max(T,T_{0})}\right|)$$

where: *T*_0_ represents the rutting critical temperature, °C; *A*, *B* represents regression coefficients.

**Figure 14:**
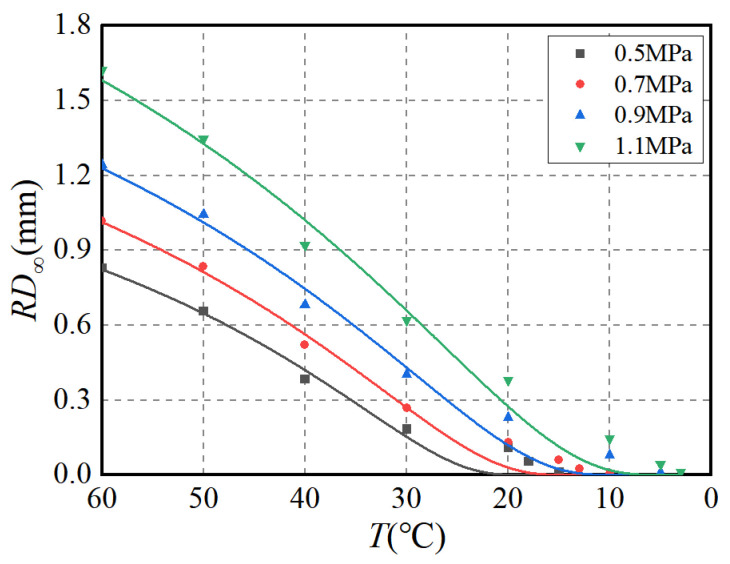
Correlation between permanent deformation and temperature of LSAM-50 asphalt mixture.

Substituting the data from Table 8 into Equation (4), as shown in Figure 14, regression analysis yields the fitted curve for the preliminary model, with relevant parameters listed in Table 9.

**Figure 15:**
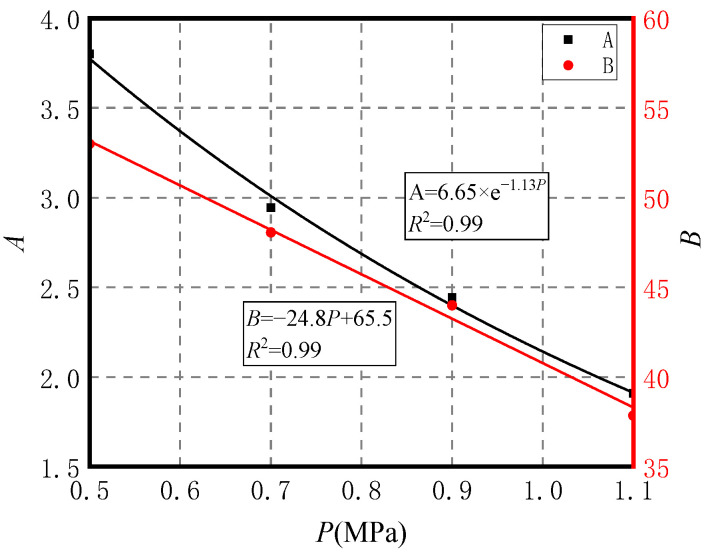
Relationship between fitting parameters and load.

According to Table 9, parameters *A* and *B* are both related to load, the functional relationship is shown in Figure 15.

Substituting the calculation formulas for the two parameters from Figure 15 into Equation (4) yields the final model for permanent deformation as a function of temperature and load, shown in Equation (5).

(5) $$RD_{\infty (T)}=6.65\times e^{-1.13P}\times EXP(-\left|\frac{-24.8P+65.5}{T_{0}-\max(T,T_{0})}\right|)$$

where *T* represents test temperature, °C; *P* represents load, MPa.

**Figure 16:**
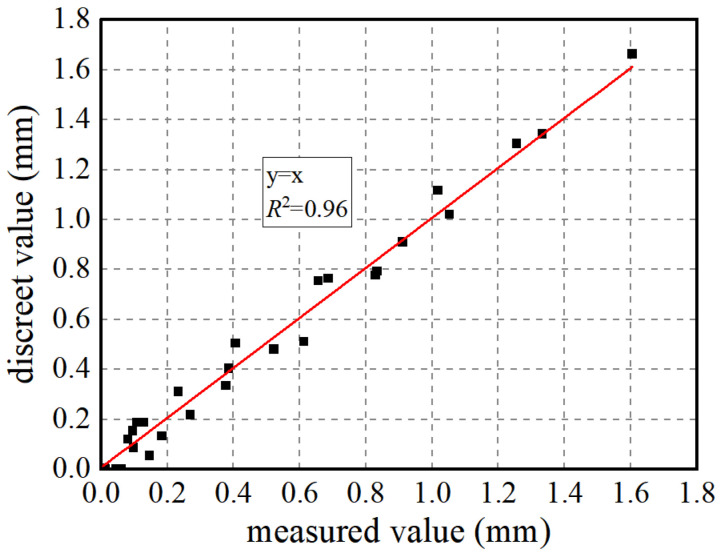
Correlation between estimated and measured permanent deformation values of LSAM-50 asphalt mixture.

Calculating the permanent deformation of the LSAM-50 asphalt mixture according to Equation (5), the correlation between the estimated values and measured values is shown in Figure 16.

From Figure 16, it can be seen that the estimated values are highly correlated with the measured values. It is recommended to use Equation (5) to predict the permanent deformation of the LSAM-50 asphalt mixture under different temperatures and loads.

### 4.2. Study on Correlation Between Permanent Deformation and High-Temperature Evaluation Indicators

Currently, the 1 h rutting depth, compressive strength, and shear strength of asphalt mixtures are commonly used to evaluate their high-temperature stability.

(1) Correlation between Permanent Deformation and 1 h Rutting Depth

The ratios of the rutting depth *RD*_N_ to the permanent deformation RD_∞_ of the LSAM-50 asphalt mixture are shown in Table 10, where 638, 1275, 1913, and 2520 cycles correspond to loading times of 15 min, 30 min, 45 min, and 60 min, respectively.

According to Table 10, when the number of load applications is small, the ratio of the rutting depth to permanent deformation of the LSAM-50 asphalt mixture at different temperatures under various loads is relatively scattered. However, as the number of loading cycles increases, the ratios tend to converge. Concurrently, the rutting deformation during the first 15 min under each load approaches 50% of the permanent deformation, indicating that the rutting depth of the mixture is largely dictated by the deformation generated during the compaction transition period. When loaded for 1 h (2520 cycles), the ratio of rutting deformation to the ultimate deformation under each load is approximately 80%. Consequently, the permanent deformation of the LSAM-50 asphalt mixture under specific conditions can be predicted using the 1 h rutting depth.

**Figure 17:**
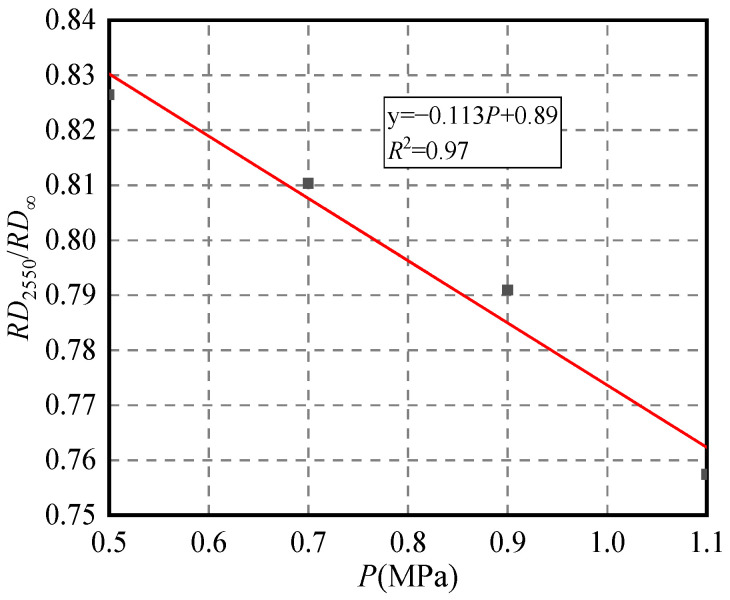
Relationship between *RD*_2520_/*RD*_∞_ of LSAM-50 asphalt mixture and load.

The ratio of 1 h rutting deformation to permanent deformation for each load is presented in Table 11 and Figure 17.

**Figure 18:**
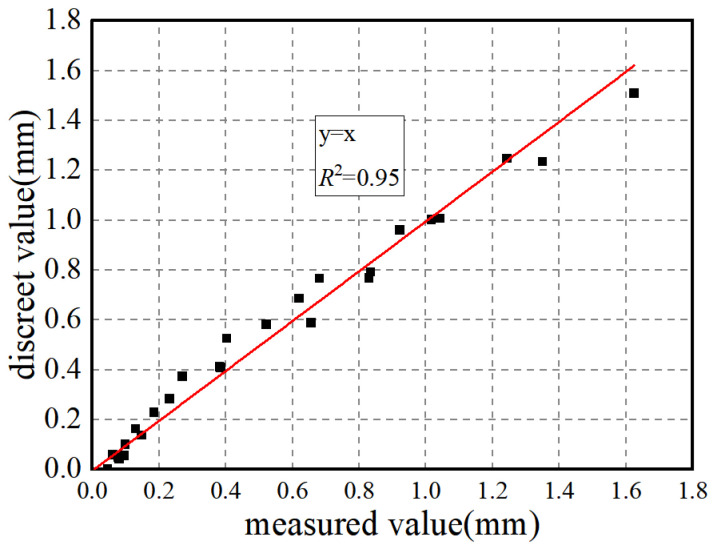
Correlation between estimated and measured permanent deformation values of LSAM-50 asphalt mixture.

Based on Figure 17, a sound linear relationship exists between *RD*_2520_/*RD*_∞_ and load *P*, as shown in Equation (6); from this, the empirical formula correlating the mixture’s permanent deformation with the 1 h rutting depth is determined, as shown in Equation (7). Calculating the permanent deformation of the LSAM-50 asphalt mixture utilizing Equation (7), the correlation between estimated and measured values is shown in Figure 18.

(6) $$\frac{RD_{2520}}{RD_{\infty }}=-0.113P+0.89$$

(7) $$RD_{\infty }=\frac{RD_{2520}}{-0.113P+0.89}$$

From Figure 18, it can be concluded that: the estimated permanent deformation values of the LSAM-50 asphalt mixture are highly correlated with the measured values. Therefore, Equation (7) can be utilized to predict the permanent deformation of the LSAM-50 asphalt mixture using the 1 h rutting depth.

(2) Correlation between Permanent Deformation and Compressive Strength

**Figure 19:**
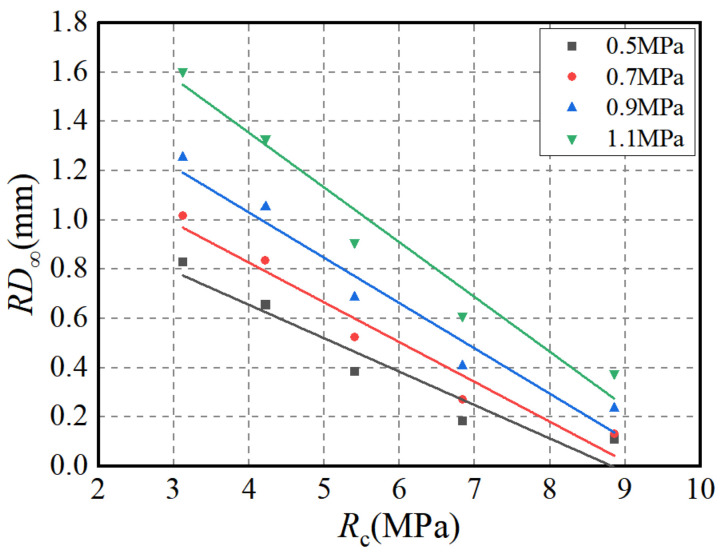
Relationship between permanent deformation of LSAM-50 asphalt mixture and compressive strength.

Uniaxial compression tests were conducted on the LSAM-50 mixture in accordance with the T0713-2000 method specified in the JTG 3410-2025 [35] standard. The compressive strength and permanent deformation of the LSAM-50 asphalt mixture under identical temperature conditions are presented in Table 12. Consequently, their correlative relationship is established as depicted in Figure 19.

According to Figure 19, a robust linear relationship exists between permanent deformation and compressive strength, as shown in Equation (8), with relevant parameters in Table 13.

(8) $$RD_{\infty }=A_{R1}R_{c}+B_{R1}$$

**Figure 20:**
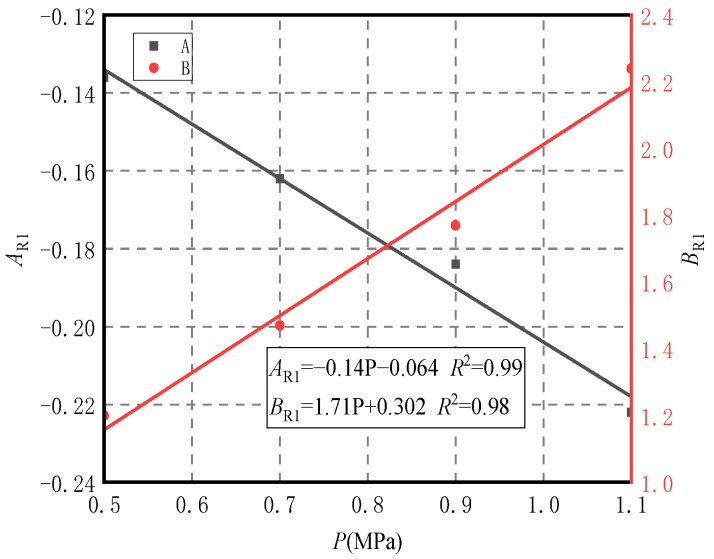
Relationship between various coefficients and load.

According to Table 13, parameters *A*_R1_ and *B*_R1_ are both related to load *P*. The relationships between the two parameters and load are established as shown in Figure 20.

**Figure 21:**
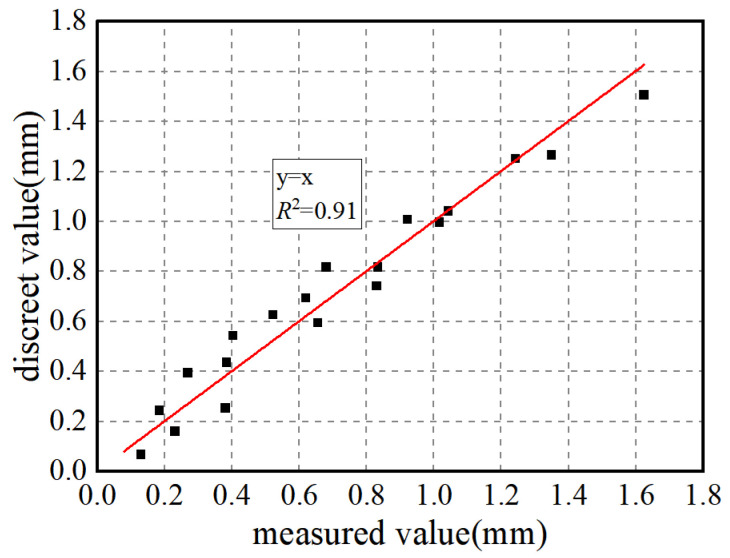
Correlation between estimated and measured permanent deformation values.

By substituting the empirical formulas for the two parameters versus load from Figure 20 into Equation (8), the empirical formula relating the permanent deformation of the LSAM-50 asphalt mixture to compressive strength is established, as shown in Equation (9). Thus, by calculating the permanent deformation of the LSAM-50 asphalt mixture utilizing load and compressive strength, the correlation between the estimated values and measured values is shown in Figure 21.

(9) $$RD_{\infty }=(-0.14P-0.064)R_{c}+1.71P+0.302$$

From Figure 21, it can be observed that: the estimated values are highly correlated with the measured values. Therefore, compressive strength and wheel load can be utilized to calculate the permanent deformation of the LSAM-50 asphalt mixture.

(3) Correlation between Permanent Deformation and Shear Strength

The fundamental cause of rutting deformation in asphalt mixtures is that the shear strength of the material is lower than the shear stress induced by the repeated action of temperature and mechanical loading. Conventionally, this shear strength is characterized by the internal friction angle (φ) and cohesion (c). Consequently, this subsection aims to determine the c and φ parameters of the LSAM-50 mixture based on uniaxial compression tests and splitting tests (the latter conducted in accordance with the T0716-2025 method specified in the JTG 3410-2025 standard [35]). The experimental results regarding the ultimate permanent deformation (RD_∞_), cohesion (c), and the tangent of the internal friction angle (tanφ) of the LSAM-50 asphalt mixture under identical temperature conditions are summarized in Table 14.

Based on this, a multiple regression analysis is performed on the permanent deformation of the LSAM-50 asphalt mixture with respect to cohesion and internal friction angle, as shown in Equation (10). Relevant parameters are provided in Table 15.

(10) $$RD_{\infty }=A_{R2}\times c+B_{R2}\times \tan\varphi +D_{R2}$$

**Figure 22:**
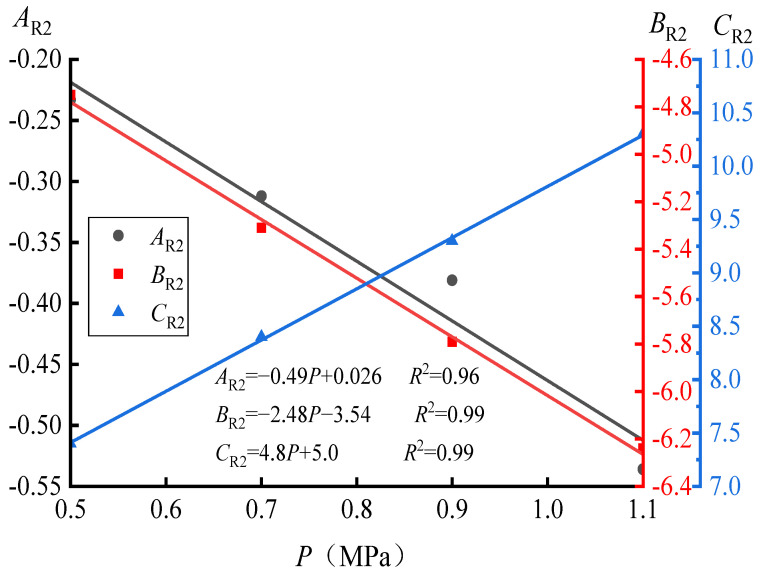
Relationship between various coefficients and load.

Based on Table 15, each coefficient in Equation (10) is a function of load P. As illustrated in Figure 22, the relationships between the respective coefficients and the load are established.

**Figure 23:**
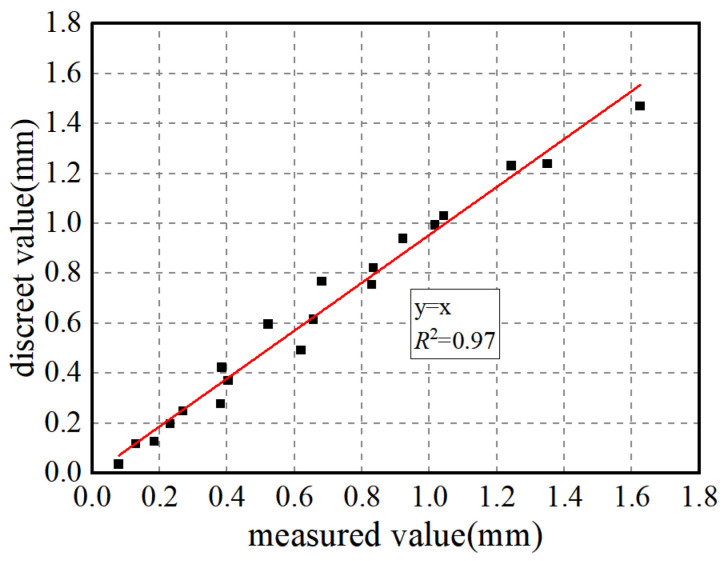
Correlation between estimated and measured permanent deformation values.

Substituting the empirical formulas for the three coefficients versus load from Figure 22 into Equation (10) establishes the empirical formula correlating the permanent deformation of the LSAM-50 asphalt mixture with shear strength, as seen in Equation (11). Consequently, by utilizing the load and shear strength parameter values *c* and *φ* to calculate the permanent deformation of the LSAM-50 asphalt mixture, the correlation between the estimated values and the measured values is shown in Figure 23.

(11) $$RD_{\infty }=(-0.49P+0.026)c+(-2.48P-3.54)\tan\varphi +4.8P+5.0$$

From Figure 23, it can be observed that: the estimated values have a high correlation with the measured values. Therefore, the shear strength parameter values *c*, *φ*, and wheel load *P* can be employed to calculate the permanent deformation of the LSAM-50 asphalt mixture.

## 5. Conclusions

(1) Both temperature and load exert a significant influence on the dynamic stability of the LSAM-50 asphalt mixture, with an evident interactive effect existing between the two factors. As temperature rises or load escalates, the dynamic stability exhibits a pronounced decline. The maximum attenuation rate occurs within the 40–50 °C interval (approaching the softening point of the asphalt), reaching 40–57%.

(2) The rutting deformation of the LSAM-50 asphalt mixture demonstrates distinct temperature and load dependency. With an increasing number of loading cycles, the rutting deformation displays a trend of initial rapid growth followed by stabilization. Furthermore, the magnitude of deformation increases significantly under elevated temperatures and heavier applied loads.

(3) During the stepwise temperature elevation and load increment processes, the rutting deformation exhibits a step-like upward growth trend. After the deformation stabilizes under lower temperature or lighter load conditions, subsequent heating or loading still induces further rutting. However, the preliminary actions of low temperature or light load impart a certain “training effect” on the rutting resistance of the pavement material.

(4) For the mixture compacted by high-temperature wheel tracking, almost no deformation is generated during the subsequent cooling process; similarly, negligible deformation occurs during the unloading process for the mixture compacted under heavy loads. Specifically, when the load is initially reduced to 0.9 MPa, the incremental deformation in this phase is a mere 0.04 mm; upon further unloading to 0.7 MPa, the incremental deformation approaches 0 mm.

(5) A permanent deformation-temperature-load dependency model for the LSAM-50 asphalt mixture was established, achieving a high correlation of 96%. Moreover, the permanent deformation exhibits a robust linear relationship with the 1-h rutting depth (R^2^ = 0.95), compressive strength (R^2^ = 0.91), and shear strength (R^2^ = 0.97), enabling rapid estimation of rutting performance through these specific metrics.

(6) This study is primarily based on macroscopic laboratory mechanical tests and has not yet incorporated the complex coupling effects of environmental factors such as moisture, temperature fluctuations, and aging. Additionally, the fundamental mechanism of the “training effect” currently lacks support from meso-scale data. Future research will integrate X-ray Computed Tomography (CT) and numerical simulations to quantitatively dissect the internal restructuring mechanisms of the thick-lift aggregate skeleton. Furthermore, there is a plan to conduct field calibration for the proposed model relying on data from actual engineering test sections, thereby enhancing its engineering applicability.

## Figures and Tables

**Table 1:** Technical indexes of asphalt.

Index		Test Method	Measured Value
Density (15 °C, g·cm^−3^)		T 0603—2025	1.041
Penetration (25 °C, 0.1 mm)		T 0604—2011	62
Ductility (10 °C, cm)		T 0605—2011	36
Softening point (°C)		T 0606—2011	46.2
After TFOT (163 °C, 5 h)	Mass loss (%)	T 0609—2011	0.11
	Ductility (10 °C, cm)	T 0605—2011	8
	Penetration ratio (25 °C, %)	T 0604—2011	64

**Table 2:** Technical indicators of coarse aggregates.

Index	Aggregate Size (mm)					
	37.5–53	19–37.5	9.5–19	4.75–9.5	2.36–4.75	0–2.36
Apparent relative density	2.803	2.769	2.748	2.732	2.730	2.734
Flakiness content (%)	2.1	8.0	6.9	12.9	/	/
Water absorption (%)	0.32	0.71	0.96	1.16	1.69	/

**Table 3:** Mineral grading of asphalt mixtures.

**Sieve size (mm)**	53	37.5	19	9.5	4.75	2.36	1.18	0.6	0.3	0.15	0.075
**Passing ratio by mass (%)**	100.0	70.0	60.0	42.0	34.0	26.0	18.0	14.0	10.0	7.5	4.5

**Table 4:** Density of wheel tracking specimens under different compaction passes.

Thickness (cm)	Number of Compaction Passes (Passes) *ρ*_B_ (g/cm^3^)							Number of Compaction Passes (Passes) *ρ*_B_/*ρ*_V_						
	24	28	32	36	40	44	48	24	28	32	36	40	44	48
12	2.360	2.454	2.543	2.557	2.562	2.565	2.568	0.93	0.96	1.00	1.00	1.01	1.01	1.01
14	2.324	2.437	2.498	2.549	2.558	2.561	2.563	0.91	0.96	0.98	1.00	1.00	1.01	1.01
16	2.294	2.391	2.444	2.500	2.548	2.556	2.558	0.90	0.94	0.96	0.98	1.00	1.00	1.00
18	2.248	2.329	2.413	2.472	2.518	2.551	2.556	0.88	0.91	0.95	0.97	0.99	1.00	1.00

**Table 5:** Two-Way ANOVA Table for Dynamic Stability.

Index	Sum of Squares of Deviations (Passes/mm^2^)	Degree of Freedom	Mean Square Fluctuation (Passes/mm^2^)	F-Value
Temperature	4.53 × 10^10^	4	1.13 × 10^10^	F*_T_* = 11.6
Load	1.87 × 10^10^	3	6.23 × 10^9^	F*_P_* = 6.4
Interaction	2.96 × 10^10^	12	2.47 × 10^9^	F*_I_* = 2.5
Error	3.89 × 10^10^	40	9.73 × 10^8^	-
Total	1.32 × 10^11^	59	-	-

**Table 6:** Deformation of LSAM-50 asphalt mixture under different temperature control modes.

Load (MPa)	Index	Variations of Indicators at Various Temperatures (mm)				
		20 °C	30 °C	40 °C	50 °C	60 °C
0.5	*RD*_S_	0.088	0.095	0.102	0.111	0.117
	*RD*_L1_	0.088	0.183	0.286	0.396	0.513
	*RD*_HW_	0.088	0.178	0.349	0.599	0.756
0.7	*RD*_S_	0.138	0.127	0.144	0.167	0.164
	*RD*_L1_	0.138	0.264	0.408	0.576	0.739
	*RD*_HW_	0.138	0.252	0.467	0.767	0.951
0.9	*RD*_S_	0.217	0.159	0.168	0.187	0.170
	*RD*_L1_	0.217	0.376	0.544	0.731	0.901
	*RD*_HW_	0.217	0.364	0.605	0.990	1.221
1.1	*RD*_S_	0.329	0.182	0.184	0.192	0.175
	*RD*_L1_	0.329	0.511	0.695	0.887	1.061
	*RD*_HW_	0.329	0.536	0.798	1.250	1.647

**Table 7:** LSAM-50 Asphalt Mixture Deformation under Different Temperature Control Modes.

Temperature (°C)	Index	Variations of Indicators at the Following Loads			
		0.5 MPa	0.7 MPa	0.9 MPa	1.1 MPa
20	*RD*_Z_	0.088	0.045	0.036	0.029
	*RD*_L2_	0.088	0.134	0.170	0.199
	RD_HZ_	0.088	0.131	0.217	0.329
30	*RD*_Z_	0.178	0.070	0.061	0.043
	*RD*_L2_	0.178	0.248	0.309	0.353
	RD_HZ_	0.178	0.252	0.364	0.536
40	*RD*_Z_	0.349	0.101	0.08	0.069
	*RD*_L2_	0.349	0.450	0.530	0.599
	RD_HZ_	0.349	0.467	0.605	0.798
50	*RD*_Z_	0.599	0.148	0.105	0.092
	*RD*_L2_	0.599	0.747	0.852	0.944
	RD_HZ_	0.599	0.767	0.990	1.250
60	*RD*_Z_	0.756	0.173	0.139	0.182
	*RD*_L2_	0.756	0.929	1.068	1.250
	RD_HZ_	0.756	0.951	1.221	1.571

**Table 8:** Growth equation of rutting deformation.

Load (MPa)	Temperature (°C)	RD_∞_ (mm)	ξ	R^2^
0.5	15	0.012	530	0.96
	20	0.109	530	0.94
	30	0.184	530	0.99
	40	0.385	530	0.99
	50	0.656	530	0.99
	60	0.829	530	0.99
0.7	10	0.006	610	0.99
	15	0.061	610	0.99
	17	0.098	610	0.98
	20	0.129	610	0.97
	30	0.269	610	0.97
	40	0.522	610	0.98
	50	0.834	610	0.98
	60	1.017	610	0.99
0.9	5	0.010	680	0.99
	10	0.081	680	0.93
	20	0.233	680	0.99
	30	0.406	680	0.98
	40	0.686	680	0.98
	50	1.052	680	0.99
	60	1.255	680	0.97
1.1	5	0.045	760	0.94
	7	0.095	760	0.99
	10	0.146	760	0.99
	20	0.377	760	0.98
	30	0.612	760	0.97
	40	0.911	760	0.98
	50	1.333	760	0.99
	60	1.604	760	0.96

**Table 9:** Fitting parameters for the permanent deformation equation.

Load (MPa)	A	B	T_0_	R^2^
0.5	3.80	53	20	0.98
0.7	2.94	48	17	0.98
0.9	2.44	44	10	0.99
1.1	1.91	38	7	0.97

**Table 10:** LSAM-50 RD_N_/RD_∞_.

Load (MPa)	Temperature (°C)	*RD*_638_/*RD*_∞_	*RD*_1275_/*RD*_∞_	*RD*_1913_/*RD*_∞_	*RD*_2520_/*RD*_∞_
0.5	15	0.414	0.621	0.746	0.829
	20	0.366	0.580	0.721	0.821
	30	0.508	0.686	0.776	0.831
	40	0.582	0.724	0.788	0.824
	50	0.575	0.721	0.787	0.825
	60	0.578	0.722	0.787	0.825
0.7	10	0.457	0.738	0.844	0.879
	15	0.387	0.587	0.710	0.786
	17	0.429	0.622	0.724	0.796
	20	0.405	0.602	0.719	0.797
	30	0.498	0.669	0.755	0.807
	40	0.557	0.702	0.768	0.807
	50	0.519	0.682	0.761	0.808
	60	0.498	0.669	0.755	0.807
0.9	5	0.498	0.664	0.747	0.797
	10	0.338	0.538	0.671	0.766
	20	0.472	0.648	0.739	0.796
	30	0.519	0.676	0.752	0.797
	40	0.542	0.689	0.757	0.797
	50	0.457	0.638	0.734	0.795
	60	0.419	0.609	0.719	0.789
1.1	5	0.230	0.408	0.551	0.741
	7	0.460	0.432	0.716	0.768
	10	0.424	0.601	0.697	0.758
	20	0.504	0.652	0.722	0.764
	30	0.500	0.650	0.722	0.764
	40	0.499	0.649	0.721	0.764
	50	0.415	0.594	0.693	0.757
	60	0.369	0.557	0.671	0.747

**Table 11:** LSAM-50 *RD*_2520_/*RD*_∞_.

Load (MPa)	LSAM-50 *RD*_2520_/RD_∞_										Mean Value
	5 °C	7 °C	10 °C	15 °C	17 °C	20 °C	30 °C	40 °C	50 °C	60 °C	
0.5	-	-	-	0.83	-	0.82	0.83	0.82	0.83	0.82	0.83
0.7	-	-	0.88	0.79	0.80	0.80	0.81	0.81	0.81	0.81	0.81
0.9	0.80	-	0.77	-	-	0.80	0.80	0.80	0.79	0.79	0.79
1.1	0.77	0.77	0.76	-	-	0.76	0.76	0.76	0.76	0.75	0.76

**Table 12:** Test results of compressive strength and permanent deformation.

Temperature (°C)	Compressive Strength (MPa)	Permanent Deformation of LSAM-50 Asphalt Mixture at the Following Loads (mm)			
		0.5 MPa	0.7 MPa	0.9 MPa	1.1 MPa
20	8.86	8.86	0.109	0.129	0.233
30	6.84	6.84	0.184	0.269	0.406
40	5.41	5.41	0.385	0.522	0.686
50	4.22	4.22	0.656	0.834	1.052
60	3.12	3.12	0.829	1.017	1.255

**Table 13:** Parameters related to the fitting equation.

**Load (MPa)**	***A*_R1_**	***B*_R1_**	**R^2^**
0.5	−0.136	1.20	0.93
0.7	−0.162	1.47	0.94
0.9	−0.184	1.77	0.94
1.1	−0.222	2.24	0.95

**Table 14:** Parameters related to the fitting equation.

*T* (°C)	*c* (MPa)	tan*φ*	Permanent Deformation of LSAM-50 Asphalt Mixture at the Following Loads (mm)			
			0.5 MPa	0.7 MPa	0.9 MPa	1.1 MPa
20	1.53	1.47	8.86	0.109	0.129	0.233
30	1.12	1.47	6.84	0.184	0.269	0.406
40	0.86	1.42	5.41	0.385	0.522	0.686
50	0.64	1.39	4.22	0.656	0.834	1.052
60	0.43	1.37	3.12	0.829	1.017	1.255

**Table 15:** Parameters Related to the Prediction Equation.

**Load (MPa)**	***A*_R2_**	***B*_R2_**	***D*_R2_**	**R^2^**
0.5	−0.233	−4.75	7.4	0.98
0.7	−0.312	−5.31	8.4	0.98
0.9	−0.381	−5.79	9.3	0.98
1.1	−0.536	−6.24	10.3	0.98

## Data Availability

The original contributions presented in this study are included in the article. Further inquiries can be directed to the corresponding author.
